# MUCOR-ADVANCE: Uniting multidisciplinary science to defeat a deadly fungus

**DOI:** 10.1371/journal.ppat.1014736

**Published:** 2026-10-28

**Authors:** Sebastian Wurster, Adam G. Stewart, Yiyou Gu, Eman G. Youssef, Teny M. John, Priya Uppuluri, Georgios Chamilos, Scott Filler, Vincent Bruno, Ashraf S. Ibrahim, Dimitrios P. Kontoyiannis, on behalf of the MUCOR-ADVANCE consortium

**Affiliations:** 1 Department of Infectious Diseases, Infection Control and Employee Health, The University of Texas MD Anderson Cancer Center, Houston, Texas, United States of America; 2 The Lundquist Institute at Harbor-University of California Los Angeles (UCLA) Medical Center, Torrance, California, United States of America; 3 David Geffen School of Medicine at UCLA, Los Angeles, California, United States of America; 4 School of Medicine, University of Crete, Voutes, Heraklion, Crete, Greece; 5 Institute of Molecular Biology and Biotechnology, Foundation for Research and Technology, Heraklion, Crete, Greece; 6 Department of Microbiology and Immunology, University of Maryland School of Medicine, Baltimore, Maryland, United States of America; 7 Institute for Genome Sciences, University of Maryland School of Medicine, Baltimore, Maryland, United States of America; University of Maryland, Baltimore, UNITED STATES OF AMERICA

## Introduction

Mucormycosis is a severe fungal infection caused by Mucorales molds that primarily affects immunocompromised individuals, including those with diabetes, cancer, and recipients of solid organ or hematopoietic cell transplants [1]. The infection can also be a devastating complication of severe trauma or burns, especially in the context of natural disasters and war-related injuries [1]. Mucormycosis is emerging worldwide, is hyper-endemic in South Asia, and gained worldwide attention when >50,000 cases of Covid-19–associated mucormycosis crippled already strained hospital systems in India [1]. The most common manifestations are invasive sino-orbital infection seen in diabetic patients, pulmonary infection in patients with hematologic cancer, and cutaneous disease, usually seen in patients with trauma [1]. Regardless of the initial manifestation, mucormycosis is highly angioinvasive and can disseminate rapidly. The average treatment cost per patient in the U.S. exceeds six figures [2]. Despite aggressive antifungal therapy with liposomal amphotericin B and/or Mucorales-active triazoles, often combined with surgical debridement, mortality rates are >50% and can approach 100% in patients with disseminated disease, persistent neutropenia, or brain involvement [1].

The significant global burden and severity of mucormycosis have led to its inclusion in both the World Health Organization “Fungal Priority Pathogens List” [3] and U.S. National Institute of Allergy and Infectious Diseases (NIAID) “Biodefense & Emerging Infectious Diseases List” [4], underscoring the urgent need for breakthroughs in translational mucormycosis research. Encouragingly, several recent discoveries offer promise for clinical translation. Herein, we highlight important milestones in mucormycosis research in the 2020s and introduce the NIAID-funded program project “MUCOR-ADVANCE”, a collaborative multi-institutional platform uniting science to defeat this deadly infection.

## What are the primary research focus areas to improve mucormycosis outcomes?

Key challenges for mucormycosis management include the delay in diagnosis, lack of prognostic risk stratification biomarkers, and limited therapeutic options, compounded by our limited knowledge of Mucorales pathogenicity and host defense. Because early treatment of the disease markedly improves therapeutic outcomes even in highly immunosuppressed hosts [5], the development of new culture-independent diagnostic modalities for early detection of mucormycosis would enable precision surveillance strategies and treatment of localized disease in high-risk patients. Further, more early culture-independent diagnostics would enable assessment of disease burden and identify early treatment failure. Prognostic models informed by a combination of clinical risk factors, routine clinical parameters, surrogates of fungal burden, and innovative immune biomarkers would facilitate the delivery of individualized, risk-adapted treatment strategies to optimize patient outcomes. Because current antifungal drug treatments of mucormycosis often fail, future combination therapies targeting elimination of the pathogen, neutralizing toxins, and boosting the host defense hold promise in improving therapeutic outcome. These combination therapies will be informed by translational research on the critical junctures of fungal virulence, host defenses, and metabolic factors. These efforts are anticipated to evolve in concert with the discovery and clinical evaluation of new Mucorales-active antifungal agents, including innovative amphotericin B formulations and antifungals with novel mechanisms of action [1,6,7].

## How will recent diagnostic advances help improve mucormycosis management?

Major issues which hinder prompt diagnosis of mucormycosis include the inability to obtain a suitable microbiological specimen, delayed results and insensitivity of conventional culture-based methods, and poor specificity of histological examination [1]. Molecular-based assays are well-positioned to address these challenges. Given the high angioinvasion and early dissemination of Mucorales, the molecular detection of circulating Mucorales DNA may be particularly promising [8]. A recently published meta-analysis quoted a pooled sensitivity and specificity of molecular testing by polymerase chain reaction (PCR) on bronchoalveolar lavage (BAL) fluid of 97.5% and 95.8%, respectively [9]. However, a high level of methodological heterogeneity (e.g., DNA extraction, gene targets, PCR platforms) results in variable diagnostic yield. Quantitative PCR assays have also been developed which may be useful in identifying severe, high-burden disease and monitoring response to therapy [10]. Such PCR-based approaches have shown promise to enable early preemptive therapy, positively affecting outcome in some patient cohorts at high risk for mucormycosis (e.g., burn patients) [11]. Although several methodological issues remain to be addressed [12], detection of cell-free fungal DNA in plasma is another potential fast and non-invasive solution to mucormycosis diagnosis, with a study citing a sensitivity and specificity of 85.1% and 92.9%, respectively [13]. The diagnostic yield increased greatly when immunosuppression and disseminated disease were present [13].

To improve accessibility and turnaround time of molecular testing, point-of-care platforms are being developed. These include loop-mediated isothermal amplification (LAMP), a cost-effective assay that does not require sophisticated equipment and showed promising performance on both clinical and preclinical specimens [14]. In addition, a lateral-flow device detecting an extracellular polysaccharide antigen specific to Mucorales fungi has recently been developed [15]. Early experience in archived biospecimens suggests sensitivity and specificity around 75%, showing potential as an affordable point-of-care test for rapid diagnosis of mucormycosis [15].

## How can novel therapeutic approaches disrupt key virulence factors of Mucorales fungi?

Recent discoveries have identified specific molecular events that allow Mucorales to bypass human defenses, offering new avenues for precision diagnostics and targeted immunotherapies. Specifically, the glucose-regulated protein 78 kDa (GRP78) receptor on endothelial cells and nasal epithelial cells and integrin/epidermal growth factor receptor (EGFR) receptor activation cascade on lung epithelial cells were identified as primary entryways for Mucorales [16–18]. The fungal spore coat proteins (CotH) bind to GRP78 and integrins, triggering host cell endocytosis and subsequent damage (Fig 1A). The CotH3/GRP78 interaction is significantly upregulated in states of hyperglycemia and ketoacidosis, providing a molecular explanation for the extreme vulnerability of diabetic patients to mucormycosis [16,19]. Translating this discovery into potential therapy, a murine monoclonal antibody and its humanized version (VX-01) targeting a conserved epitope among CotH proteins universally present in Mucorales was shown to protect against mucormycosis in preclinical models by blocking Mucorales-mediated tissue invasion (Fig 1B) [20]. Importantly, targeting angioinvasion is likely to enhance antifungal drug delivery to the site of infection and potentially explains the *in vivo* synergy seen with VX-01 and antifungal drugs [20]. Beyond passive immunization, these surface proteins may also represent attractive candidates for recombinant vaccines.

**Fig 1:**
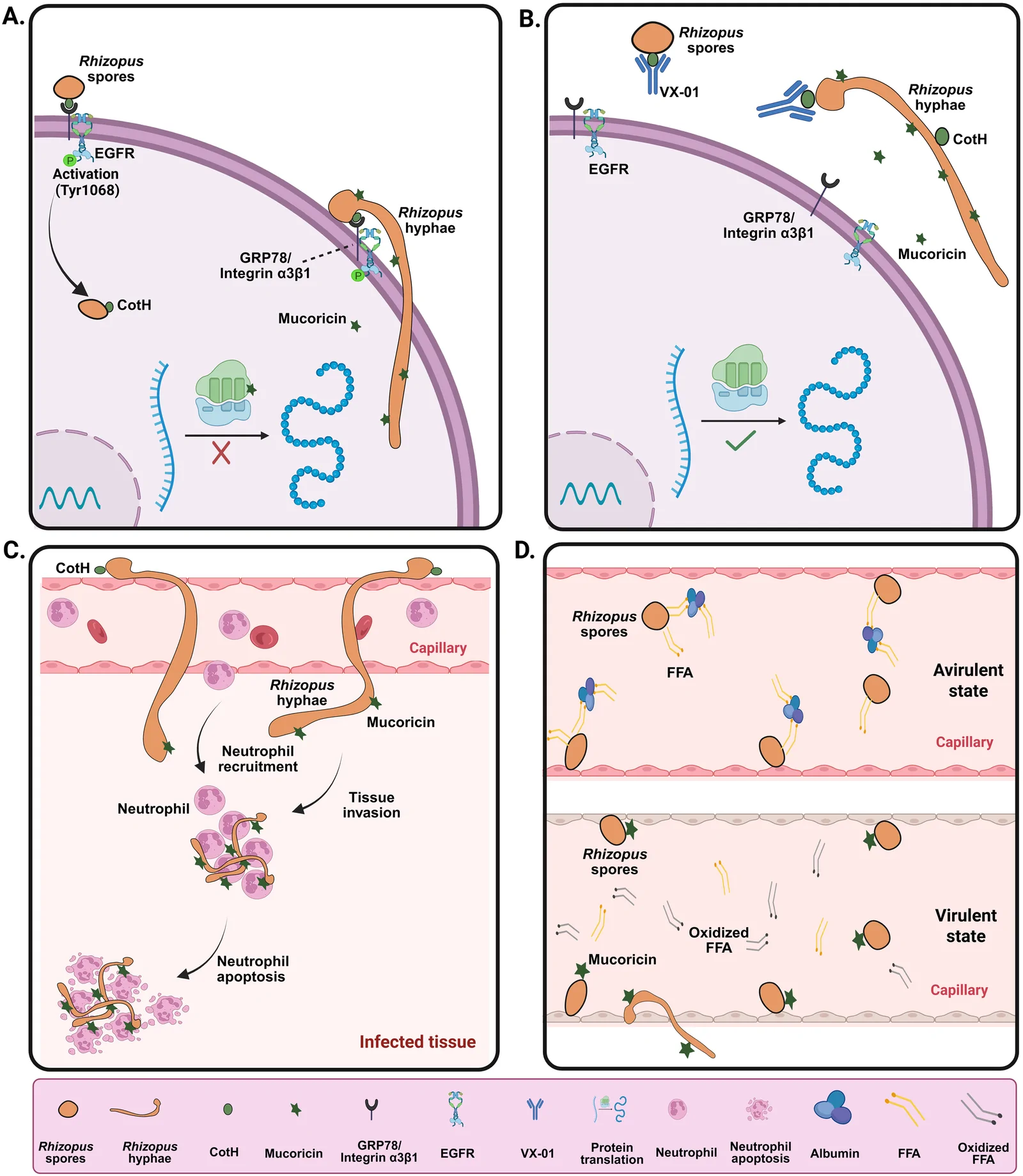
Current understanding of Mucorales pathogenesis. **(A)** Following germination, CotH ligands interact with host receptors, including GRP78 and integrin α3β1, promoting fungal adherence, endocytosis, and tissue invasion. Concurrently, mucoricin enters host cells and inhibits protein translation, contributing to host cell dysfunction and tissue injury. **(B)** VX-01, a monoclonal antibody targeting CotH ligands, blocks CotH–host receptor interactions and prevents CotH-mediated fungal invasion. **(C)** Tissue invasion triggers recruitment of neutrophils to sites of infection; however, exposure to mucoricin induces neutrophil apoptosis, thereby impairing antifungal host defense. **(D)** Proposed model of albumin-mediated host defense during *Mucorales* infection. Albumin-bound free fatty acids (FFAs) inhibit mucoricin activity and contribute to maintenance of an avirulent fungal state. During infection, oxidation of FFAs reduces their protective activity, permitting mucoricin activity and promoting a virulent state. Schematics are not drawn to scale; the blood vessel spaces in panels C and D are depicted at different scales for clarity.

Moreover, the discovery of mucoricin, a ricin-like toxin, has revealed the mechanism behind the rapid vascular permeability and extensive tissue necrosis that often renders antifungal drugs unable to reach the site of infection [21]. Additionally, mucoricin can bind to the Mucorales cell wall and create a “molecular trap” that induces neutrophil apoptosis and disrupt effective responses by neutrophil clusters (so-called “neutrophil swarming”), leading to an invasive infection (Fig 1C) [22]. Preclinical studies suggest that anti-toxin therapy may slow tissue necrosis, preserve the microvasculature, and improve antifungal drug delivery. These effects could also enhance protective immune responses against invasive infection [21,22].

## And what about the host side?

A recent landmark study identified albumin as a master regulator of a novel metabolic host defense mechanism against Mucorales that prevents invasive disease by modulating fungal pathogenicity [23]. Albumin elicits selective antifungal activity against Mucorales by shuttling, solubilizing, protecting, and releasing physiological free fatty acids (FFAs), which are taken up by fungal cells and inhibit protein synthesis and virulence [23] (Fig 1D). Notably, preexposure of germinating spores to albumin enhances neutrophil swarming and prevents invasive disease by inhibiting mucoricin expression [22,23]. In patients with very low albumin levels, oxidation of albumin-bound FFAs results in loss of their antifungal properties, allowing unrestricted Mucorales growth [23]. Consequently, hypoalbuminemia emerged as an independent predictor of mortality in mucormycosis patients across three global patient cohorts [23]. This discovery suggests that immuno-metabolic interventions, such as restoration of albumin levels in high-risk patients, could enhance host resilience against Mucorales [23].

Hypoxia-inducible factor 1-alpha (HIF1α) was identified as another centerpiece of the host-pathogen interface, playing two opposing roles in airway epithelial cells during Mucorales infection [24]. While promoting fungal invasion into host cells, infection-induced activation of HIF1α also contributes to proinflammatory responses during mucormycosis [24]. Thus, blockade of HIF1α may attenuate both fungal invasion of airway epithelia and exuberant inflammation. However, more preclinical evidence is needed in diverse host backgrounds, as HIF1α may contribute to both protective immunity and hyperinflammation, and was required for protection in other pulmonary mold infection models [25].

Furthermore, immunostimulatory agents are being evaluated to help immunocompromised hosts in clearing the infection. Cytokines (IFN-γ) and hematopoietic growth factors (G-CSF and GM-CSF) are already used as salvage immunotherapy in patients with severe fungal infections [1]. Notably, recent preclinical research showed that prophylactic GM-CSF may prevent mucoricin-induced apoptotic death of neutrophils, enhance “neutrophil swarming”, and thereby protect against invasive mucormycosis [22].

Moreover, both preclinical and clinical data indicate that immuno-inhibitory checkpoint pathways are upregulated rapidly after infection with Mucorales and contribute to a dysfunctional immune environment with induction of exhaustion markers, suppression of T-cell signaling, and impairment of innate immune effector responses [26–28]. Blocking these pathways with checkpoint inhibitors, a proven type of cancer immunotherapy, can enhance host defense and promote fungal elimination in experimental mucormycosis [26,27]. Despite likely publication bias, this approach has been supported by several case reports of salvage use in patients whose mucormycosis was irresponsive to antifungal therapy [29]. Preclinical data suggests that early combination therapy with antifungals and immunomodulators may be most effective in intercepting infection-induced immune dysregulation [26], re-emphasizing the convergence of rapid diagnostics and application of reliable prognostic markers to identify patients who might benefit from early immunostimulatory therapy (Fig 2). In the future, tailored host-targeted interventions (i.e., immune augmentation versus dampening of injurious inflammation) are expected to rely on the integration of multiple immunological parameters that collectively capture dynamic changes in immune defense, aided by evolving omics technologies, advanced data science models, and artificial intelligence [30].

**Fig 2:**
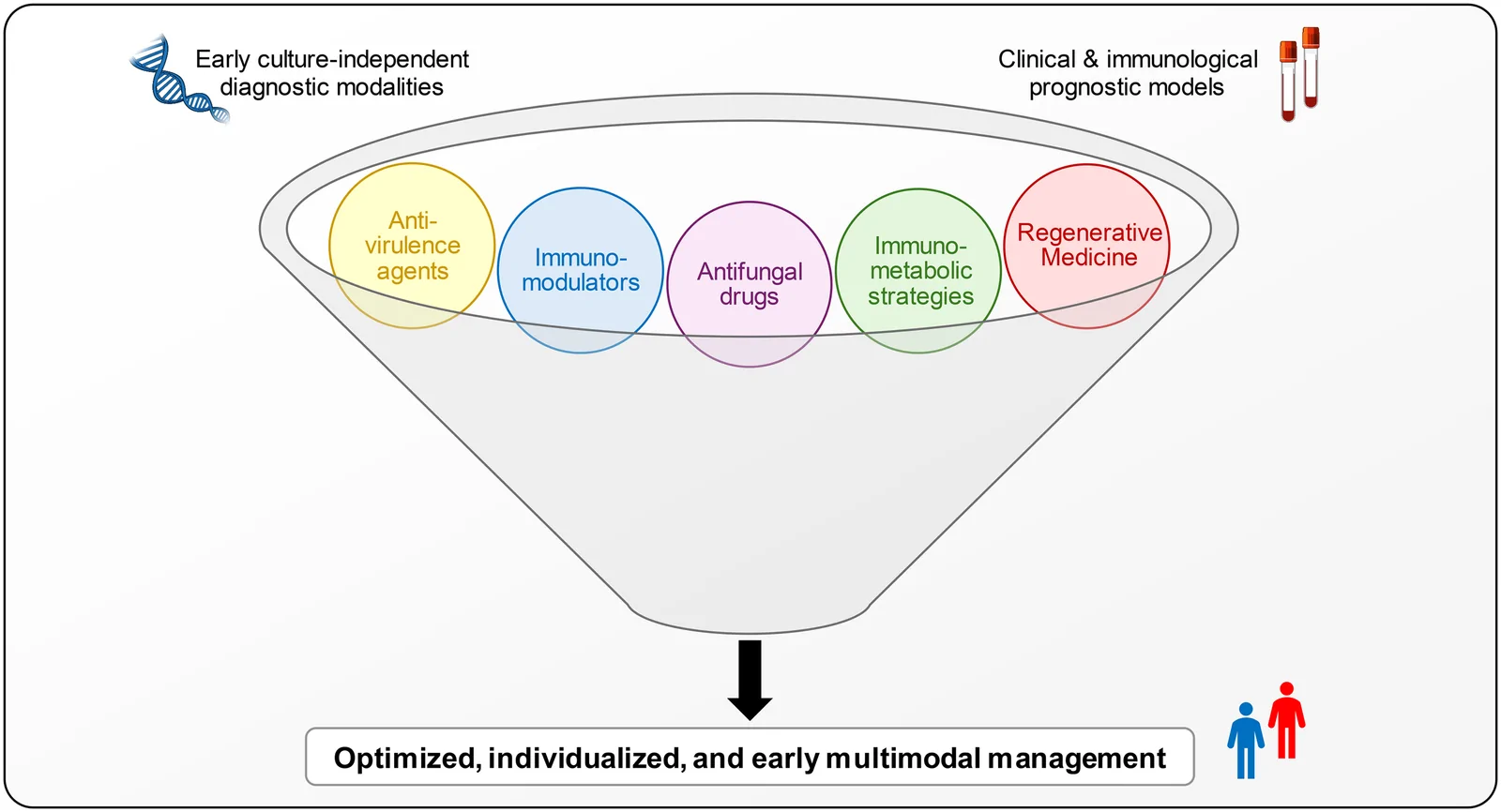
Conceptual framework for translational impact through personalized precision management.

## How will MUCOR-ADVANCE bring it all together?

MUCOR-ADVANCE (https://www.mucoradvance.com) is a multidisciplinary research unit dedicated to transforming the “bench-to-bedside” pipeline for mucormycosis management. Moving away from traditional “silo-based” research, the NIAID program project grant (P01) mechanism affords a coordinated, multi-institutional research platform. MUCOR-ADVANCE includes synergistic research projects focused on *Fungal Biology* (The Lundquist Institute), *Host Biology and Regenerative Medicine* (The Lundquist Institute), and *Translational Immunobiology* (MD Anderson Cancer Center). A Genomics/Transcriptomics Core at the University of Maryland provides access to advanced sequencing and bioinformatics capabilities for data-driven discoveries and biomarker development. Moreover, a Clinical Core at MD Anderson Cancer Center prospectively collects patient data, Mucorales isolates, and diverse host-derived biospecimens from mucormycosis patients and relevant control groups (pulmonary aspergillosis, bacterial pneumonia, and high-risk patients with no infections).

In addition to preclinical validation in mouse models mirroring various risk factors for mucormycosis, including diabetic ketoacidosis, glucocorticosteroid immunosuppression, neutropenia, and active leukemia, such biorepositories will be essential to inform, prioritize, and cross-validate diagnostic, therapeutic, and risk stratification strategies. In concert with the broader mucormycosis research community and scientific societies like the recently founded International Immunotherapy Society for Fungal Diseases [31], MUCOR-ADVANCE ultimately aims to inch closer to early and individualized precision management of mucormycosis through coordinated advances in discovery science, risk stratification, diagnosis, prognostic biomarkers, pathogen- and host-targeted therapeutics, and regenerative medicine (Fig 2). Beyond mucormycosis, this multidisciplinary approach may serve as a blueprint for future studies of other rare mycotic diseases and opportunistic infections.
